# Global prevalence of post-miscarriage anxiety, depression, and stress: a systematic review and meta-analysis

**DOI:** 10.7189/jogh.15.04245

**Published:** 2025-09-26

**Authors:** Asha Shetty, Alwin Issac, Sanjay Dhiraaj, Vijay VR, Latha Thimappa, Deepthy Balakrishnan, Bhola Nath, Shruti Sinha, Suyash Singh, Prabakar Mishra, Kurvatteppa Halemani

**Affiliations:** 1 College of Nursing, All India Institute of Medical Sciences, Bhubaneswar, India; 2 Department of Anaesthesiology, Sanjay Gandhi Post Graduate Institute of Medical Sciences, Lucknow India; 3 College of Nursing, All India Institute of Medical Sciences, Raebareli, India; 4 College of Nursing, All India Institute of Medical Sciences, Bibinagar, India; 5 Department of Obstetrics & Gynaecology, All India Institute of Medical Sciences, Bhubaneswar, India; 6 Community and Family Medicine, All India Institute of Medical Sciences, Raebareli, India; ^7^Department of Psychiatry, All India Institute of Medical Sciences, Raebareli, India ^8^Department of Neurosurgery, All India Institute of Medical Sciences, Raebareli, India; 9 Department of Biostatistics & Health Informatics, Sanjay Gandhi Post Graduate Institute of Medical Sciences, Lucknow, India

## Abstract

**Background:**

Mental disorders, ranked as the sixth leading cause of disability-adjusted life years in 2017, present significant challenges to healthcare systems. Anxiety and depression are frequently reported among pregnant women, particularly after experiencing a miscarriage. We aim to evaluate the global prevalence of anxiety, depression, and stress in women following a miscarriage.

**Methods:**

We searched electronic databases, including PubMed (MEDLINE), Cochrane, Cumulative Index to Nursing and Allied Health Literature, EMBASE, Web of Science, Scopus, and ClinicalKey, for studies published in English from January 1995 to December 2024. We adhered to the Cochrane Collaboration guidelines and reported it using the PRISMA 2020 statement. Anxiety, depression, and stress were the primary outcomes we assessed.

**Results:**

In this systematic review and meta-analysis, we included 29 studies with 35 375 participants. Participants’ age ranged from 18 to 45 years. We found that 32.5% of women experienced anxiety, 30.1% depression, and 33.6% stress within six weeks following a miscarriage. Notably, the prevalence of these mental health disorders was higher in low- and middle-income countries.

**Conclusions:**

Our findings underscore the impact of mental health on overall well-being, particularly after a miscarriage. Healthcare professionals need to acknowledge the importance of mental health during the six weeks following a miscarriage. Early identification and treatment of mental disorders are crucial for mitigating their effects on women's mental health and improving outcomes in future pregnancies.

**Registration:**

PROSPERO: CRD42024578605.

Mental disorders are a major contributor to the global burden of non-fatal diseases, with anxiety and depression being the most frequently reported [1]. While some mental disorders may be resolved without treatment, others require extensive management. Anxiety involves feelings of worry and fear related to specific situations, and a certain amount of anxiety can motivate individuals to complete tasks promptly [2]. In contrast, depression is marked by persistent sadness and hopelessness, often leading to thought blocking, negativity, and altered perceptions. Various personal and environmental risk factors influence both anxiety and depression. Additionally, trauma to the head, substance abuse, and hormonal changes can impact mood and behaviour. Psychiatric issues affect both genders but are more prevalent in women, particularly during and after pregnancy [3–5].

Pregnancy involves numerous physiological changes, including hormonal fluctuations that can impact the mental well-being of pregnant and postpartum women. The first trimester is particularly crucial due to the heightened risk of abortion and miscarriage [6–8]. Early pregnancy loss is a traumatic experience for women and their families, with spontaneous abortions typically occurring before 24 weeks and often going unnoticed in the first eight weeks [9]. Recurrent miscarriages may be associated with chromosomal abnormalities or maternal factors [10,11]. Therefore, providing adequate counselling for patients and their families, along with social support and timely management, is essential [12].

Pregnancy is a proud journey, anticipating a new family member, but the sudden loss of a pregnancy can cause intense emotional trauma and shock [13]. The impact of pregnancy loss is typically more profound for the mother than the father, often linked to anxiety, depression, and stress. Furthermore, mental disorders were the sixth leading cause of disability-adjusted life years in 2017, posing significant challenges for the healthcare system [14]. Although there have been notable advancements in diagnosing and treating maternal health, mental health – particularly following abortion or pregnancy loss – often remains neglected, yet it is essential for future pregnancies [15]. While some studies report on the prevalence of mental health issues after pregnancy loss, there is no synthesis of evidence on this topic [16]. We highlight the importance of integrating mental healthcare for women who have experienced a miscarriage, as these concerns are often overlooked, increasing the risk of mental health issues. Developing guidelines to address these psychological challenges is crucial.

Anxiety, depression, and stress are common psychiatric disorders following traumatic events, yet they are often neglected. Depression often manifests in individuals who have experienced uncertain loss or damage, particularly in pregnant and postpartum women due to hormonal changes. It is especially prevalent among women with a history of miscarriage. Symptoms can range from mild to severe, and many women may experience long-term effects. Therefore, we included these variables in our analysis, aiming to assess the global prevalence of anxiety, depression, and stress among women following miscarriage.

## METHODS

In this systematic review and meta-analysis, we aimed to synthesise the evidence concerning the global prevalence of anxiety, depression, and stress among women following miscarriage. We adopted the Cochrane Collaboration guidelines to carry out this systematic review and meta-analysis, and reported it using the PRISMA 2020 statement [17]. We prospectively registered the study protocol in PROSPERO (CRD42024578605).

For the search strategy, we used a combination of MeSH terms, with key phrases including anxiety, depression, stress, and prevalence, structured using Boolean operators in Patient, Intervention, Comparison, and Outcome format: (anxiety OR afraid OR fear OR anxious) AND (depression OR worthlessness OR helplessness OR hopelessness) AND (stress OR distress OR strain OR deprived child) AND (pregnancy loss OR abortion OR miscarriage OR early pregnancy loss) AND (prevalence OR cross-sectional study OR observational study OR cohort study). We searched online databases, including PubMed, ClinicalKey, Embase, Scopus, Web of Science, CINAHL, and Cochrane, for original studies published in English from 1 January 1995 to 30 June 2024, without any restriction on the study's location. We snowballed the reference lists of the included articles for identification of further relevant articles and documented the search history.

The inclusion criteria were observational studies whose participants were women within six weeks post miscarriage, studies that adopted standardised measurement tools for assessing stress, anxiety, and depression, and studies that reported cut-off scores for anxiety, depression, and stress among participants. We excluded studies that evaluated the efficacy of various interventions for stress, anxiety, and depression among participants.

The first and the second reviewer (KH and IL) independently screened the articles, resolving any disagreements through team discussion with the third reviewer (AI). Any further disagreements were resolved in consultation with the fourth reviewer (AS). We imported the search results into Rayyan software [18], which uses the ‘check for duplicates’ tool to remove duplicate entries. Using the search strategy, we found 34 971 articles from the electronic databases, identifying four additional articles through manual searches. We excluded a total of 4578 duplicates. We screened the titles and abstracts of the remaining 30 397 articles, and, following the inclusion criteria, excluded additional 30 269 articles. We screened the full texts of the remaining 128 articles based on the eligibility criteria, and removed another 99 articles. The reasons for excluding articles were participants having other comorbid conditions, differences in primary outcomes, and insufficient statistical data. Finally, we included 29 articles. The study selection process is depicted in

We assessed the methodological quality and risk of the articles using a modified Newcastle-Ottawa Scale [19]. This scale consists of five criteria: sample representativeness, sample size, response rate, tool name and cut-off scores, and statistical details. Each item carries one point, with studies scoring more than three considered low risk.

Two authors (KH and AS) thoroughly screened and independently reviewed the eligible studies, resolving any disagreements through discussion with the third author (AI). We summarised the extracted data under headings including author name, year of publication, country, sample size, study period, criteria, instrument, and outcomes.

We manually entered and coded the data from the studies in Microsoft Excel 2021 (Microsoft Corporation, Washington, USA), before transferring them to Stata, version 17 (Stata Corp LP, College Station, USA). We assessed the heterogeneity among studies using the *I*^2^ test, categorising the *I*^2^ values as high (>75%), medium (50–75%), and low (<50%) [20]. We employed a random effects model due to the observed heterogeneity. We calculated the effect sizes (ESs) with 95% confidence intervals (CIs) using the Metaprop and Metan commands. We conducted subgroup analyses based on differences in anxiety, depression, and stress across geographical regions and income levels.

## RESULTS

**Figure 1:**
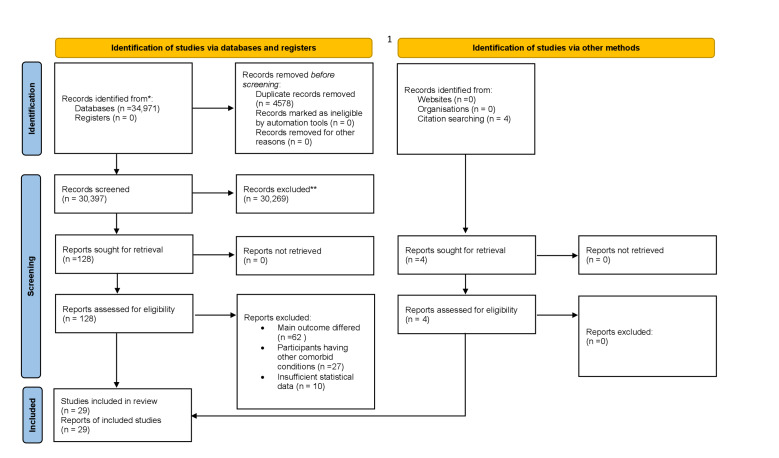
PRISMA flow diagram for study selection.

We identified 29 articles, including a total of 35 375 women with a history of miscarriage [21-49]. Of these, 19 studies reported anxiety disorders, including seven studies from Asia [23,24,27,34,42,43,50], 11 studies from Europe [21,22,26,28,29,31,35,46–48,51], and one study from Africa [49]. Similarly, eleven studies reported depression, with seven from Europe [21,22,26,28,29,31,35,46–48,51], fourteen from Asia [23-25,27,34,36,38,41–45,50], three from Africa [39,48,49], and one from Australia [32]. Additionally, seven studies from Europe [21,22,28,33,40,47,51], two from Africa [48,49], and one from Asia [52] reported stress (**Figure 1**, **Table 1**, **Table 2**).

**Table 1:** Characteristics of the included studies

Study, year, country	Study design	Sample size, median (range)	Age, x̄ (SD)	Instruments (COS)	Study criteria	Time of data collection	Study outcome
Farren et al., 2020, UK [21]	Cross-sectional	492 (426–1098)	32 (4)	HADS (11)	Data was obtained through email from eligible women with a history of pregnancy loss.	Four weeks	About 29% of stress, 24% of anxiety, and 11% of depression were reported after miscarriage. The symptoms reduced over time.
Farren et al., 2022, UK [22]	Cohort	737 (487–116)	34 (5)	HADS (>11)	The women who experienced pregnancy loss in the last month were included. Data was collected through email	Four weeks	About 33% of women experienced anxiety,18% depression and 37% stress after pregnancy loss. Family support reduced the symptoms.
Lee et al., 1997, Hong Kong [23]	Cross-sectional	150 (150–209)	31.3 (6.2)	SCID (>50%)	Data were collected from social media from individuals who were hospitalised.	Before six weeks	Most women experience depression (12%) and anxiety (1.3%). Compared to other countries, Hong Kong reported lower symptoms.
Sham et al., 2010, Hong Kong [24]	Cohort	169 (169–204)	31 (3.1)	GHQ-12 (>8)	Women with a history of pregnancy loss were invited. Data were directly obtained from patients.	Four weeks	About 10% reported depression, and 1.2% anxiety after pregnancy loss. The risk of psychiatric problems was younger age, infertility and a history of depression.
Lok et al., 2010, China [25]	Longitudinal	280 (280–395)	32.6 (5.7)	GHQ-12 (>8)	Participants were recruited from affiliated hospitals, and data were obtained from social media.	Six weeks	Most women experienced stress (25%) and depression (26.8%). The level of psychological symptoms reduced over time.
Cumming et al., 2007, UK [26]	Longitudinal	406 (273–686)	31.2 (NR)	HADS (>11)	The data was collected from participants who enrolled in three Scottish hospitals.	Six weeks	About 10% of women reported depression, and 28.3 % anxiety after miscarriage. Similarly, 12.4% of male partners reported anxiety and 4% depression.
Wang et al., 2023, China [27]	Cross-sectional	267 (267–663)	33.4 (3.5)	BDI (19–63), STAI (10–29), PSS (20–56)	Women with pregnancy loss who attended the clinic at Shengjing Hospital of China Medical University were included.	Six weeks	About 13.1% reported depression, and 56.6% anxiety after abortion. However, adequate social support, actively seeking health help and identifying risk factors are essential.
Kuhlmann et al., 2023, Germany [28]	Cross-sectional	122 (122–225)	Women: 35.0 (4.5) Men: 36.1 (5.5)	IES-R	Data were collected from participants with a history of miscarriage between March 2019 and October 2020.	Six	Compared to male partners (3.9%), females reported higher stress (13.7% ). The occurrence of symptoms increased among women with recurrent miscarriage.
Mendes et al., 2023, Portugal [29]	Cross-sectional	113 (113–864)	34.8 (4.9)	HADS (>11)	Participants with a history of pregnancy loss in the first 20 weeks of gestation, who were >18 years, were included.	More than four weeks	About 39.1% reported anxiety, and 13.8% depression within a month. However, family support decreased the incidence of these symptoms.
Boersma et al., 2014, Netherlands [30]	Cohort	92 (37–129)	26.58 (6.5)	CES-D (>15)	Women having an induced abortion were included. Data were directly obtained from participants.	Two to three weeks	About 30% of women reported depression following an induced abortion. Mental health should be considered after an induced abortion in the future.
Akdag et al., 2019, Turkey [31]	Cross-sectional	60 NR	NR	HADS (>11)	Women with a history of abortion were administered sociodemographic, depression, anxiety, and stress scales.	Six weeks	Most participants experienced anxiety (61.7%) and depression (85.0%). Social, family, and friend support helps to reduce these symptoms.
Taft & Watson, 2008, Australia [32]	Cohort	9327 (9327–14 776)	22–27*	CES-D (>15)	The data were obtained from participants who were seeking treatment after a miscarriage.	Six weeks	About 30% of women reported depression. Counselling and emotional support decreased these symptoms.
Kolte et al., 2015, Denmark [33]	Cross-sectional	301 (301–1813)	35 (NR)	MDI (26–50)	Women with >3 miscarriages between 2010–13 were included. Data was gathered through social media.	Six weeks	About 8.6% women had depression, and 41.2% stress after miscarriage. Psychological support and guidance may improve general health.
Gao et al., 2020, China [34]	Cross-sectional	278 (166–278)	31.8 (4.2)	EPDS (13–30)	Women with a history of recurrent abortion, between September 2016 and May 2017, were included.	Six weeks	About 45% reported anxiety, and 37.0% depression after miscarriage. Social support and counselling reduced the duration of symptoms,
Jacob et al., 2017, Germany [35]	Cohort	12 158 (1823–12 158)	31.6 (5.5)	NR	Women with spontaneous abortion between January 2007 and December 2015 were included.	Six weeks	Most participants reported depression and anxiety. Spontaneous abortion increased the psychiatric disorders.
Zhang et al., 2022, China [36]	Cross-sectional	253 (64–253)	34 (NR)	PHQ-9 (15–27)	Women with a history of miscarriage enrolled between April and October 2021 in the tertiary hospitals were included.	Six weeks	The occurrence of stress and depression is higher in Chinese women. It is essential to evaluate mental health, especially those at high risk.
Wang et al., 2021, China [50]	Case-control	1132 (1132–2558)	32.6 (3.9)	SDS (7–10)	Women with ≥2 pregnancy losses between 2017 and 2019 were included.	Six weeks	The prevalence of anxiety (28.7%) and depression (48.6%) is often seen in women after pregnancy loss. These symptoms increased the risk of subsequent miscarriage.
Azin et al., 2020, Iran [38]	Cross-sectional	130 (59–130)	31.53 (5.11)	BDI (19–63)	Women with spontaneous abortion for >2 times within six-month intervals were included.	Six weeks	About 40.8% of women reported depression. Depression is negatively associated with sexual life. Psychiatric disorders increased with multiple abortions.
Mutiso et al., 2018, Kenya [39]	Cross-sectional	173 (62–182)	29.4 (5.6)	EPDS (13–30)	Women who had experienced a miscarriage, either spontaneous or induced, were included.	Two to three weeks	About 34.1% reported depression after two weeks of abortion. Self-harm was often (33.1%) among these women.
Bialek et al., 2024, Poland [40]	Cross-sectional	161 (80–190)	NR	PSS (20–56)	Women with a history of pregnancy loss and seeking treatment were included. Data were collected through a social platform.	Six weeks	The sense of basic hope increased significantly after three months of miscarriage, and the intensity of stress decreased. Appropriate support can minimise the risk of post-traumatic stress disorder.
Chen et al., 2020, Taiwan [41]	Cross-sectional	78 (78–158)	32–35*	PSS (20–5)	Women with a history of abortion enrolled between October 2014 and July 2015 were included. Data were collected through email.	Six weeks	Women with miscarriage reported higher stress, anxiety and depression than their male partners. Health professionals need to develop supportive interventions to reduce psychological disorders.
Qu et al., 2021, China [42]	Prospective longitudinal	166 (NR)	NR	EPDS (13–30)	Women with a history of recurrent miscarriage and who were willing to give consent were included.	Six weeks	Anxiety and depression are higher in women with miscarriage. Social support is an essential buffer against anxiety and depression.
Koly et al., 2023, Bangladesh [43]	Cross-sectional	240 (141–240)	NR	PHQ-9 (15–27), GAD-7 (15–27)	Women with a history of spontaneous abortion from July 2020 to December 2021 were included.	Six weeks	The majority of the women experienced depression (77.50%) and anxiety (58.75%) after miscarriage. Health education and counselling reduced the risk, especially for women living in urban and slum areas.
Sa’D Basha et al., 2020, Jordan [44]	Cross-sectional	200 (119–200)	33.1 (6.3)	PHQ-9 (15–27), GAD-7 (15–27)	Women with abortion history enrolled between June 2018 and November 2019 in Jordan were included.	Six weeks	About 19.5% reported anxiety and 22.5% depression after miscarriage. Social support and awareness reduce the occurrence of disease.
Moafi et al., 2021, Iran [45]	Cross-sectional	185 (NR)	54	EPDS (13–30)	Women with spontaneous abortion enrolled in Kowsar teaching hospital, Qazvin, Iran, from 2015–16 were included.	Six weeks	Most women experienced depression (54%), and gestational age at the time of abortion predicted depression. Spiritual support reduced the psychological symptoms in women with spontaneous abortion.
Gashi & Blakaj, 2016, Kosovo [46]	Cross-sectional	122 (28–43 093)	28 (5.6)	EPDS (13–30)	Women who were willing to take part in the study were included. Written informed consent was obtained from eligible participants.	Six weeks	Most frequent psychological symptoms were sleep disorders (22.13%), repentance (25.40%), anger (36%), feelings of guilt and shame (27.4%), anxiety (29.5%), and depression (30.32%).
Kukulskienė & Žemaitienė, 2022, Lithuania [47]	Cross-sectional	839 (280–839)	33.34 (5.46)	EPDS (13–30)	Women with a history of multiple abortions were enrolled in the study.	Four weeks	About 59.1% reported anxiety, 48.9% depression, and 44.7% stress following pregnancy loss.
Pershad et al., 2022, Uganda [48]	Cross-sectional	1254 (708–1254)	20–29*	ACASI (>50%)	Data was collected from hospital records and patients of Kenya, Malawi, Mozambique, and Uganda.	Six weeks	About 56.5% reported anxiety and stress after pregnancy loss. Anxiety was associated with poor socioeconomic status and literacy.
Eleje et al., 2024, Nigeria [49]	Prospective case-control	47 (10–47)	18–45*	DASS-21 (19–24)	The eligible participants with a history of pregnancy loss were included. Data was collected from social media using the DASS-21 scale.	Six weeks	Women with previous pregnancy loss demonstrated higher mental disorders than women without a history of pregnancy loss. Women who experience repeated miscarriages are at higher risk of psychological problems.

ACASI – Audio computer-assisted self-interviews, BDI – Beck Depression Inventory, BDI-II – Beck Depression Inventory-II, COS – cut of score, EDS – Edinburgh Depression Scale, EPDS – Edinburgh Postnatal Depression Scale, GHQ-12 – General Health Questionnaire, HADS – Hospital Anxiety and Depression Scale, IES-R – Impact of Events Scale-Revised, MCS-A – Multi-Country Survey on Abortion-related morbidity, MPSSS – Multidimensional Perceived Social Support Scale, NR – not reported, PSQI – Pittsburgh Sleep Quality Index, PSS – Perceived Stress Scale, SAI – State-Anxiety Inventory, SAS – Self-rating Anxiety Scale, SCID – Structured Clinical Interview for DSM-III-R, SD – standard deviation, SDS – Self-rating Depression Scale, STAI – State-Trait Anxiety Inventory, TOPFA – termination of pregnancy for foetal anomaly, WHO – World Health Organization, x̄ – mean

*Values presented as a range.

**Table 2:** Quality appraisal of the included studies using the modified Newcastle-Ottawa Scale

Study, year, country	Study design	Presentiveness >70%	Sample size >250	Response rate >70%	Appropriate tool with cut-off scores	Detailed results do not require further calculations	Total score*	Study quality
Farren et al., 2020, UK [21]	Cross-sectional	1	1	1	1	1	5/5	Low risk
Farren et al., 2022, UK [22]	Cohort	1	1	1	1	1	5/5	Low risk
Lee et al., 1997, Hong Kong [23]	Cross-sectional	1	1	1	0	1	4/5	Low risk
Sham et al., 2010, Hong Kong [24]	Cohort	1	1	1	0	1	4/5	Low risk
Lok et al., 2010, China [25]	Observational	1	1	1	1	1	5/5	Low risk
Cumming et al., 2007, UK [26]	Prospective	1	1	1	1	1	5/5	Low risk
Wang et al., 2023, China [27]	Cross-sectional	1	1	1	1	1	5/5	Low risk
Kuhlmann et al., 2023, Germany [28]	Cross-sectional	1	1	1	1	0	4/5	Low risk
Mendes et al., 2023, Portugal [29]	Cross-sectional	1	1	1	1	0	4/5	Low risk
Boersma et al., 2014, Netherlands [30]	Cohort	1	1	1	1	1	5/5	Low risk
Akdag et al., 2019, Turkey [31]	Cross-sectional	1	1	1	1	1	5/5	Low risk
Taft & Watson, 2008, Australia [32]	Cohort	1	1	1	1	1	5/5	Low risk
Kolte et al., 2015, Denmark [33]	Cross-sectional	1	1	1	1	1	5/5	Low risk
Gao et al., 2020, China [34]	Cross sectional	1	1	1	1	1	5/5	Low risk
Jacob et al., 2017, Germany [35]	Cohort	1	1	1	1	1	5/5	Low risk
Zhang et al., 2022, China [36]	Cross-sectional	1	1	1	1	1	5/5	Low risk
Wang et al., 2021, China [50]	Case-control	1	1	1	1	1	5/5	Low risk
Azin et al., 2020, Iran [38]	Cross-sectional	1	1	1	1	1	5/5	Low risk
Mutiso et al., 2018, Kenya [39]	Cross-sectional	1	1	1	1	1	5/5	Low risk
Bialek et al., 2024, Poland [40]	Cross-sectional	1	1	1	1	1	5/5	Low risk
Chen et al., 2020, Taiwan [41]	Cross-sectional	1	1	1	1	1	5/5	Low risk
Qu et al., 2021, China [42]	Prospective	1	1	1	1	1	5/5	Low risk
Koly et al., 2023, Bangladesh [43]	Cross-sectional	1	1	1	1	1	5/5	Low risk
Sa’D Basha et al., 2020, Jordan [44]	Cross-sectional	1	1	1	1	0	4/5	Low risk
Moafi et al., 2021, Iran [45]	Cross sectional	0	1	1	1	1	4/5	Low risk
Gashi & Blakaj, 2016, Kosovo [46]	Cross-sectional	1	1	1	1	0	4/5	Low risk
Kukulskienė & Žemaitienė, 2022, Lithuania [47]	Cross-sectional	1	1	1	1	1	5/5	Low risk
Pershad et al., 2022, Uganda [48]	Cross-sectional	1	1	1	1	1	5/5	Low risk
Eleje et al., 2024, Nigeria [49]	Case-control	1	1	1	1	1	5/5	Low risk

*Scores of ≥3 are considered high-quality studies.

The sample size of the included studies ranged from 47 [49] to 14 776 [32], with the average age of participants being between 18 and 45 years. The instruments used to measure the prevalence of anxiety, depression, and stress included the Hospital Anxiety and Depression Scale, General Health Questionnaire, Edinburgh Postnatal Depression Scale, Beck Depression Inventory, Perceived Stress Scale, Impact of Events Scale-Revised, Edinburgh Depression Scale, State-Anxiety Inventory, Pittsburgh Sleep Quality Index, Self-rating Anxiety Scale, Self-rating Depression Scale, Audio computer-assisted self-interviews, and Structured Clinical Interview for DSM-III-R.

### Prevalence of depression among women after miscarriage

We found that 30.75% of women with a history of miscarriage experienced depression symptoms within the first six weeks (ES = 30.7; 95% CI = 23.1–38.3, *P* = 0.001; *I*^2 =^ 99.6%).

**Figure 2:**
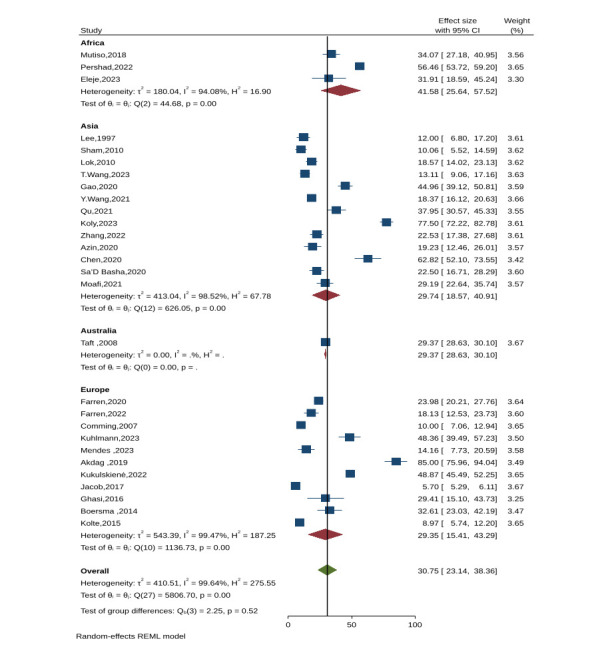
Prevalence of depression within six weeks following a miscarriage in the continental region.

On a continental level, Africa reported the highest prevalence of depressive symptoms at 41.58% (ES = 41.5; 95% CI = 25.6–57.5, *P* = 0.001, *I*^2^ = 94.08%) [39,48,49], followed by Australia at 29.37% (ES = 29.3; 95% CI = 28.6–30.1, *P* = 0.001) [52], Europe at 29.3% (ES = 29.3; 95% CI = 15.4–43.2, *P* = 0.001; *I*^2^ = 99.4%) [21,22,26,28,29,31,35,46–48,51], and Asia at 29.74% (ES = 29.7; 95% CI = 18.5–40.9, *P* = 0.001; *I*^2^ = 98.5%) [23–25,27,34,36,38,41–45,50] (**Figure 2**).

### Prevalence of anxiety among women after miscarriage

**Figure 3:**
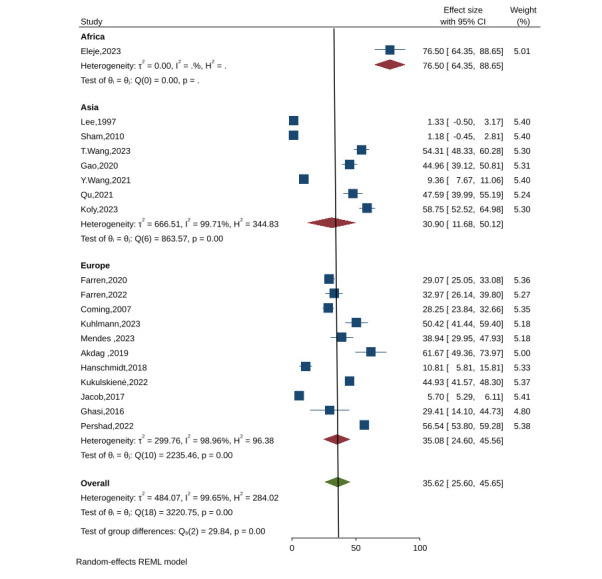
Prevalence of anxiety within six weeks following a miscarriage in the continental region.

We examined 19 studies on the prevalence of anxiety among women after miscarriage. Of these, 11 studies from Europe reported an anxiety prevalence of 35.0% (ES = 35.0; 95% CI = 24.6–45.5, *P* = 0.001; *I*^2^ = 98.9%) [21,22,26,28,29,31,35,46–48,51], while seven studies from Asia indicated a lower prevalence of 30.9% (ES = 30.9; 95% CI = 11.6–50.1, *P* = 0.00; *I*^2^ = 99.71%) [23,24,27,34,42,43,50]. Overall, global data showed that 35.6% of women experienced anxiety following miscarriage (ES = 35.6; 95% CI = 25.6–45.6, *P* = 0.001; *I*^2^ = 99.65%) (**Figure 3**).

### Prevalence of stress after miscarriage

We assessed 10 studies on the prevalence of stress among women after miscarriage. Results indicated that 33.5% of women in Europe reported stress (ES = 33.55; 95% CI = 18.4–48.7, *P* = 0.00; *I*^2^ = 98.5%) [21,22,28,33,40,47,51], followed by 45.0% in Africa (ES = 45.0; 95% CI = 21.1–69.0, *P* = 0.00; *I*^2^ = 92%) [48,49], and 13.1% in Asia (ES = 13.1; 95% CI = 9.0–17.1) [52]. Overall, global data showed that 33.66% of women reported anxiety after miscarriage (ES = 33.6; 95% CI = 21.4–45.9, *P* = 0.001; *I*^2^ = 98.65%) (Figure S1 in the **Online Supplementary Document**).

### The prevalence of anxiety, depression, and stress based on gross national product per capita

#### The prevalence of depression in low-, middle-, and high-income countries

According to the World Bank, countries are classified as high-, middle-, and low-income [53]. Among the identified articles, 15 studies reported that 26.1% of women in high-income countries experienced depression after pregnancy loss (ES = 26.1; 95% CI = 15.6–36.6, *P* = 0.00; *I*^2^ = 99.7%) [21–24,26,28–33,35,45]. Similarly, nine studies indicated a 36.0% prevalence of depression in middle-income countries (ES = 36.0; 95% CI = 21.5–50.5, *P* = 0.00; *I*^2^ = 98.7%) [25,27,34,41–43,46,50,54], while four studies reported a 36.5% prevalence in low-income countries (ES = 36.57; 95% CI = 21.7–51.3, *P* = 0.00; *I*^2^ = 96.0%) [39,44,48,49] (Figure S2 in the **Online Supplementary Document**).

#### The prevalence of anxiety in middle- and high-income countries

We assessed 19 studies that examined the prevalence of anxiety among women after miscarriage. Of these, nine studies reported that 24.1% of women in high-income countries experienced anxiety (ES = 24.1; 95% CI = 11.0–37.3, *P* = 0.00; *I*^2^ = 99.6%) [21–24,27,29,35,47,51]. In contrast, eight studies indicated that 41.0% of women in middle-income countries experienced anxiety (ES = 41.0; 95% CI = 28.6–53.5, *P* = 0.00; *I*^2^ = 97.5%) [26,28,31,34,42,43,46,50], while two studies reported a prevalence of 65.6% in low-income countries (ES = 65.6; 95% CI = 46.1–85.0, *P* = 0.00; *I*^2^ = 89.8%) [48,49] (Figure S3 in the **Online Supplementary Document**).

#### The prevalence of stress in middle- and high-income countries

Seven studies from high-income countries reported a prevalence of anxiety of 33.5% (ES = 33.5; 95% CI = 18.4–48.7, *P* = 0.00; *I*^2^ = 98.5%) [21,22,28,33,40,47,51]. One study from a middle-income country reported an anxiety prevalence of 13.1% (ES = 13.1; 95% CI = 9.0–17.1) [52], while two studies from low-income countries reported 45.0% (ES = 45.0; 95% CI = 21.1–69.0, *P* = 0.00; *I*^2^ = 92.0%) [48,49] (Figure S4 in the **Online Supplementary Document**).

#### Subgroup analysis of standardised tools

We conducted a subgroup analysis of the data collection tools to reduce heterogeneity among the studies. Studies used various standardised instruments. Five studies employed the Hospital Anxiety and Depression Scale (ES = 42.5; 95% CI = 30.7–54.9, *P* = 0.00; *I*^2^ = 93.7%) [21,22,27,29,31]. Similarly, five studies used the Edinburgh Postnatal Depression Scale (ES = 37.5; 95% CI = 30.4–44.5, *P* = 0.00; *I*^2^ = 84.2%) [34,39,45,47]. Additionally, three studies used the Patient Health Questionnaire-9 (ES = 40.8; 95% CI = 4.9–76.7, *P* = 0.00; *I*^2^ = 98.56%). The remaining scales were used in individual studies. Overall, the subgroup analysis reported an ES of 38.6 (95% CI = 31.0–46.2, *P* = 0.00; *I*^2^ = 99.0%) (Figure S5 in the **Online Supplementary Document**).

#### Galbraith plots

The Galbraith plot is a graphical representation that illustrates study-specific ESs and their precisions, as well as the overall ES, while also helping to detect potential outliers. The plot features two horizontal lines: the green line represents the reference line, indicating no effect, while the red line is the regression line. The slope of the red line reflects the overall ES and the standardised log risk ratio for each study. Circles below the green line indicate an increased risk. However, we reported no studies below the reference line (green line) in this analysis. Our meta-analysis revealed that most circles fell within the shaded region, except for two studies, which suggest that the studies were located within the 95% CI. The Galbraith plot concluded that two out of the 29 studies fell outside the shaded region, indicating considerable heterogeneity among the ESs (Figures S6 and S7 in the **Online Supplementary Document**).

## DISCUSSION

In this systematic review and meta-analysis, we present the first comprehensive overview of global data on anxiety, depression, and stress among women following miscarriage. Mental health is critical during the antenatal and postnatal periods. Miscarriage often leads to a significant emotional impact that requires attention. While many individuals experience improvement in the months that follow, it’s common for some psychological concerns to linger. Proactively screening for depression and anxiety in patients after a miscarriage is essential for their ongoing well-being and recovery [55]. Miscarriage is one of the most traumatic experiences, often leading to adverse outcomes in subsequent pregnancies. Many women face short- and long-term psychological effects of miscarriage, which frequently go unrecognised by family and healthcare professionals.

We analysed data from peer-reviewed articles published in high-impact journals using robust statistical methods. Our findings indicate that African countries reported the highest rates of depression (45.4%) and stress (56.4%) following miscarriage. Factors contributing to mental health issues in these women include family burden, number of abortions, hormonal differences, genetic predisposition, and environmental influences [56]. Furthermore, European countries exhibited higher anxiety rates (35.08%) compared to Asian countries (29.08%). The findings suggest that prevalence rates are not solely determined by national wellness or literacy levels; in fact, more developed countries reported higher rates of mental disorders than less developed ones [57]. However, most research has been conducted in high-income countries, where study quality tends to be superior. This could result in underreporting prevalence rates in lower-income nations. Therefore, further research in middle- and low-income countries is essential to address these gaps [58].

Recent studies indicate a rising incidence of mental disorders following miscarriage, highlighting a neglect of women's concerns in healthcare settings [59,60]. These issues are often overlooked, increasing the risk of mental health problems and long-term psychiatric disorders, which can significantly impact future pregnancies and place a burden on families. Early screening and intervention strategies are crucial to addressing these challenges [61,62].

Our results indicated that a significant number of participants experienced stress (33.6%), anxiety (32.5%), and depression (30.1%) following miscarriage. Approximately half of the women encountered at least one mental disorder post miscarriage [63,64]. Therefore, professional rehabilitation, cognitive therapy, and family-centred care are essential. Healthcare professionals, especially physicians and midwives, should prioritise emotional support, appropriate counselling, clear communication, and informed decision-making to assist couples in coping with miscarriage [65,66]. Ensuring the privacy of women after such events is also crucial [67]. Mental disorders may differ based on the type of miscarriage, with women typically experiencing greater distress in the first six weeks. This highlights the importance of early identification and treatment to reduce adverse mental health outcomes [68], as it can also help mitigate complications in future pregnancies. Furthermore, no randomised controlled trials have focussed on non-pharmacological interventions for women after miscarriage, highlighting the need for well-designed, double-blinded studies in this vulnerable group [69].

Culture, race, and ethnicity play a significant role in mental health, influencing both the diagnosis and treatment of psychiatric disorders. Healthcare professionals need to consider the cultural backgrounds and customs of families, as this can help to better understand their perceptions of miscarriage. Due to social stigma, families may hesitate to discuss these sensitive issues, making it essential to understand cultural practices in providing adequate care [70].

The prevalence of psychiatric disorders is often given more attention in developed countries. However, assessing their occurrence can be challenging due to the lack of national data in many regions. Mental health disorders tend to be more prevalent in developed countries, partly due to changes in diagnostic criteria and increased awareness of miscarriages, which have led to more diagnoses. Furthermore, government policies, particularly in developed countries, play a crucial role in encouraging the reporting of psychiatric disorders following pregnancy loss [71].

In 2017, mental disorders were ranked as the sixth leading cause of disability-adjusted life years, presenting significant challenges to healthcare systems. Many women experience both short- and long-term psychological effects after a miscarriage, which are often unrecognised by family members and healthcare professionals. Miscarriage is a highly traumatic event that requires immediate attention [72].

While current practices provide tailored antenatal and intranatal care, they often fall short in addressing postnatal care, particularly mental health after pregnancy loss, which can impact future pregnancy outcomes. The lack of a robust support system increases the risk of mental health disorders in women who have experienced miscarriage. Therefore, establishing guidelines to address these concerns should be an integral part of obstetric care. Additionally, the development of cognitive support strategies, focus group discussions, unique measuring tools, and the establishment of clear diagnostic criteria are essential.

Our study is the first systematic review and meta-analysis on this subject, using data from peer-reviewed articles in high-impact journals. We employed robust statistical methods and conducted subgroup analyses based on income levels and geographic locations. Our review article has several limitations. First, most studies originated from developed countries, with only a few from middle- and low-income nations, which limited our ability to generalise the global prevalence. Additionally, as the authors are only proficient in English, articles in other languages could not be included. Lastly, we did not consider grey literature, which might have introduced publication bias.

## CONCLUSIONS

Mental disorders present unique challenges, particularly for women following miscarriage. Early diagnosis and treatment can reduce psychiatric morbidity and support women in future pregnancies. Healthcare professionals, including midwives, should be aware of the early signs of mental disorders to implement appropriate interventions. Additionally, the development of cognitive support strategies, focus group discussions, unique measuring tools, and the establishment of clear diagnostic criteria are essential steps forward.

## Additional material


Online Supplementary Document
