# Supplementary material for: Global prevalence of post-miscarriage anxiety, depression, and stress: a systematic review and meta-analysis
Source: J Glob Health. 2025 Sep 26;15:04245. doi: 10.7189/jogh.15.04245 (PMC12467481; doi:10.7189/jogh.15.04245)
Supplement: Online Supplementary Document [file jogh-15-04245-s001.pdf]

Supplement to: Shetty A, Isaac A, Dhiraaj S, Vijay VR, Thimappa L, Balakrishnan D, Nath B, Sinha S, Singh S, Mishra P, Halemani K. Global prevalence of post miscarriage anxiety, depression, and stress: a systematic review and meta-analysis. J Glob Health. 2025;15:04245.

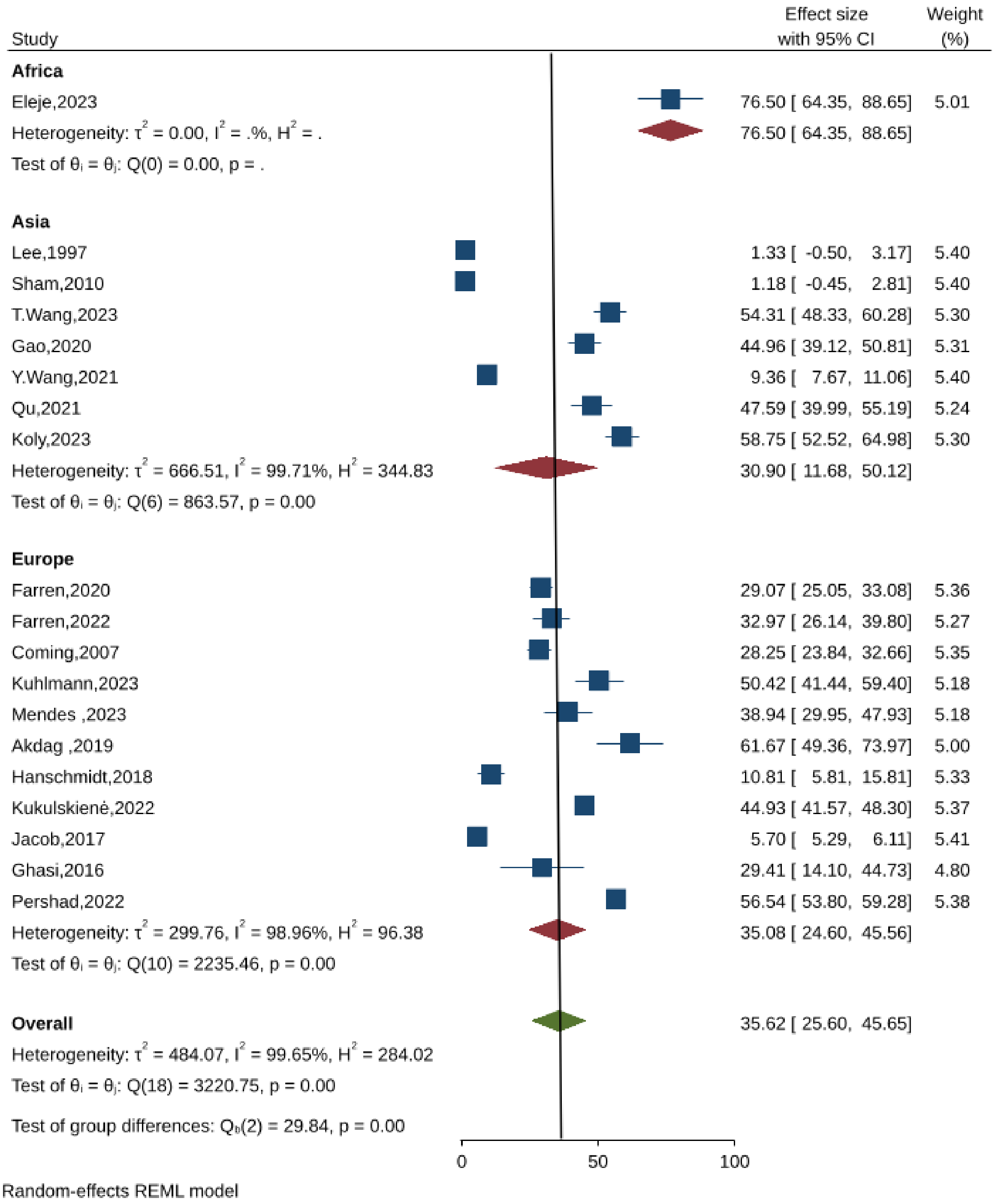

Figure S1. The prevalence of stress within six weeks following a miscarriage in the continental region.

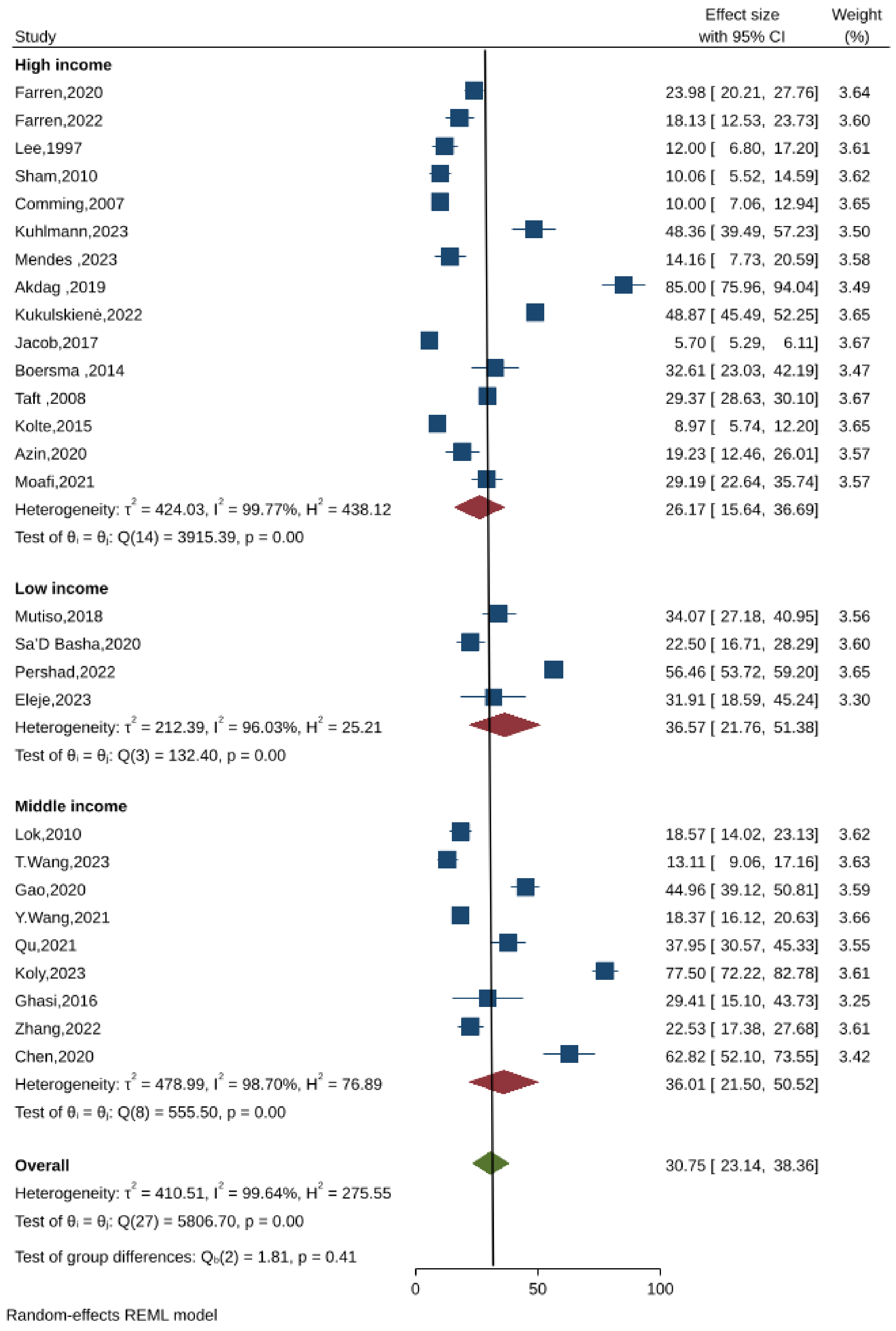

Figure S2. The prevalence of depression within six weeks following a miscarriage in the continental region.

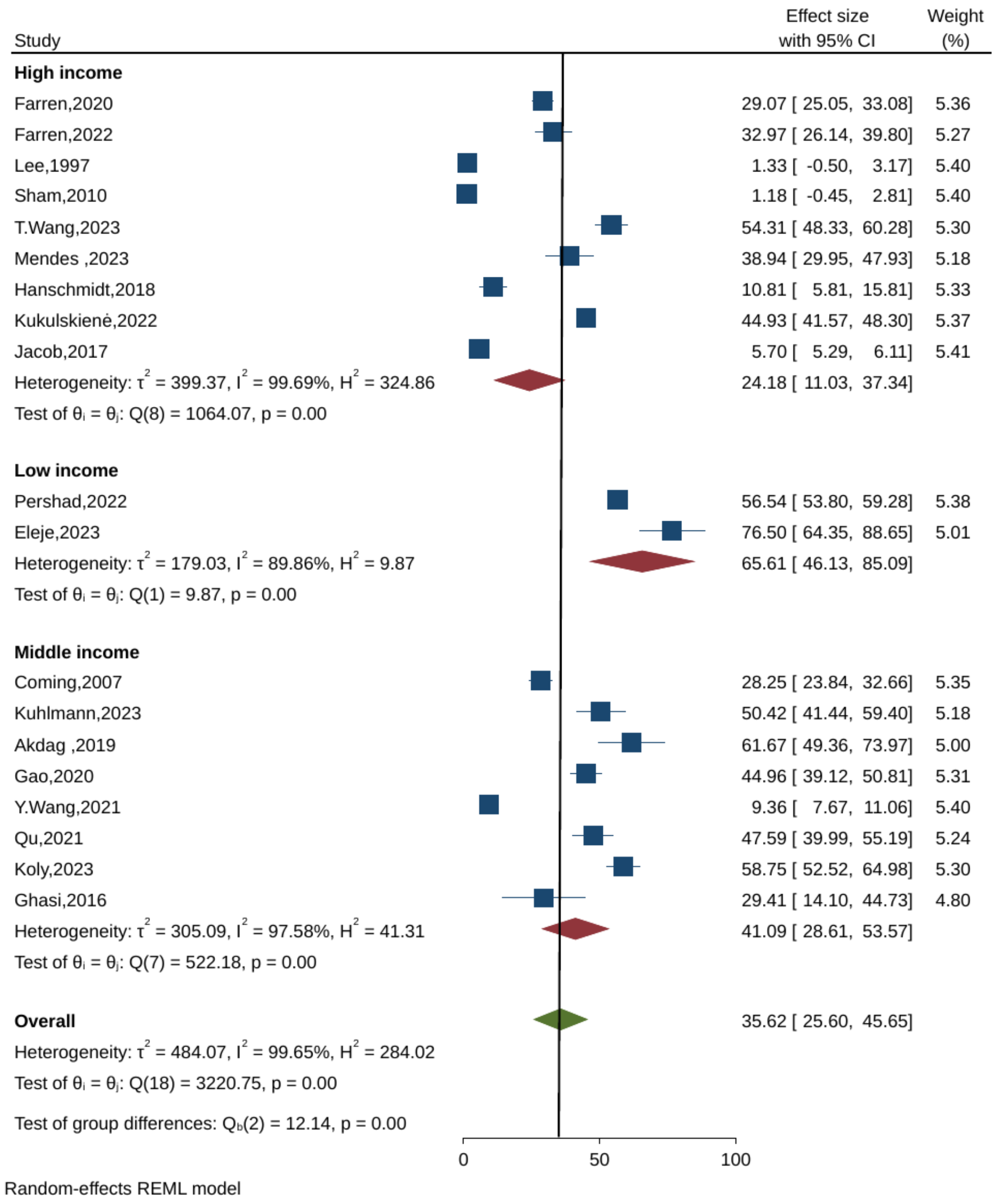

Figure S3. The prevalence of anxiety within six weeks following a miscarriage in the continental region.

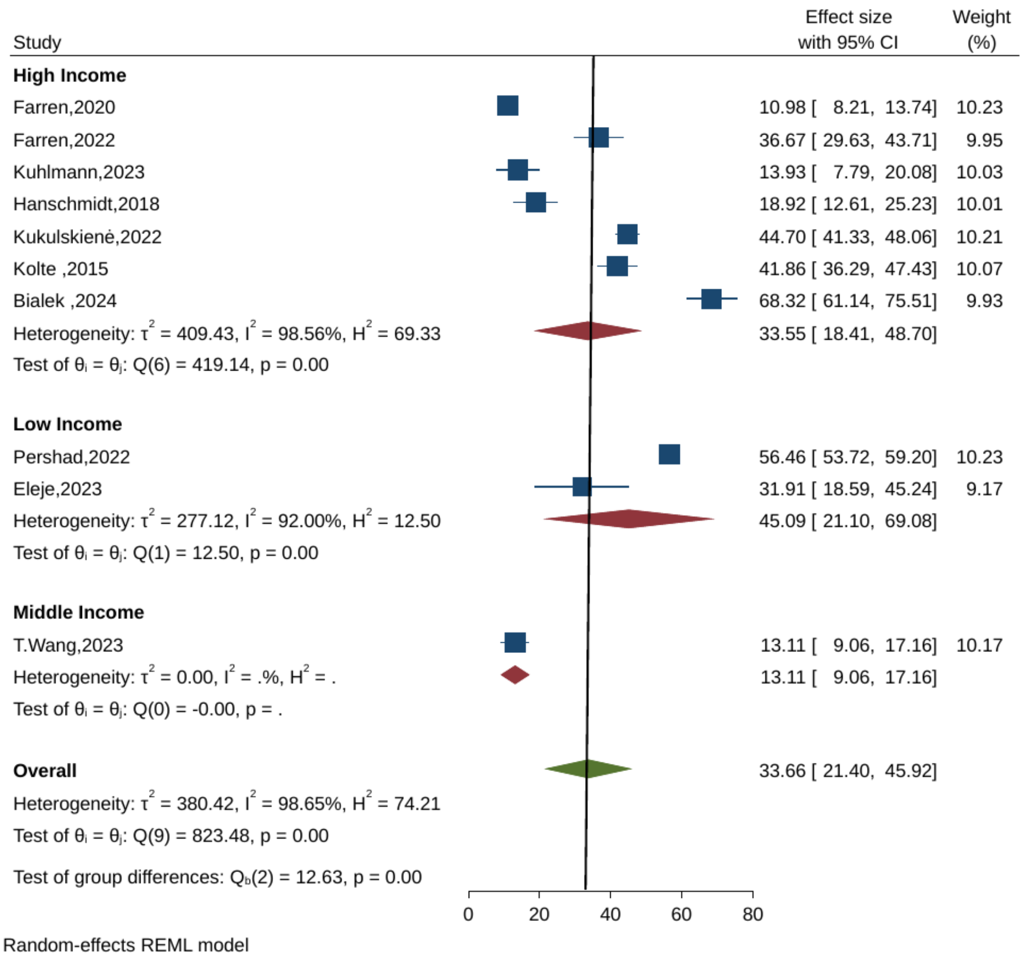

Figure S4. The prevalence of stress within six weeks following a miscarriage in the continental region.

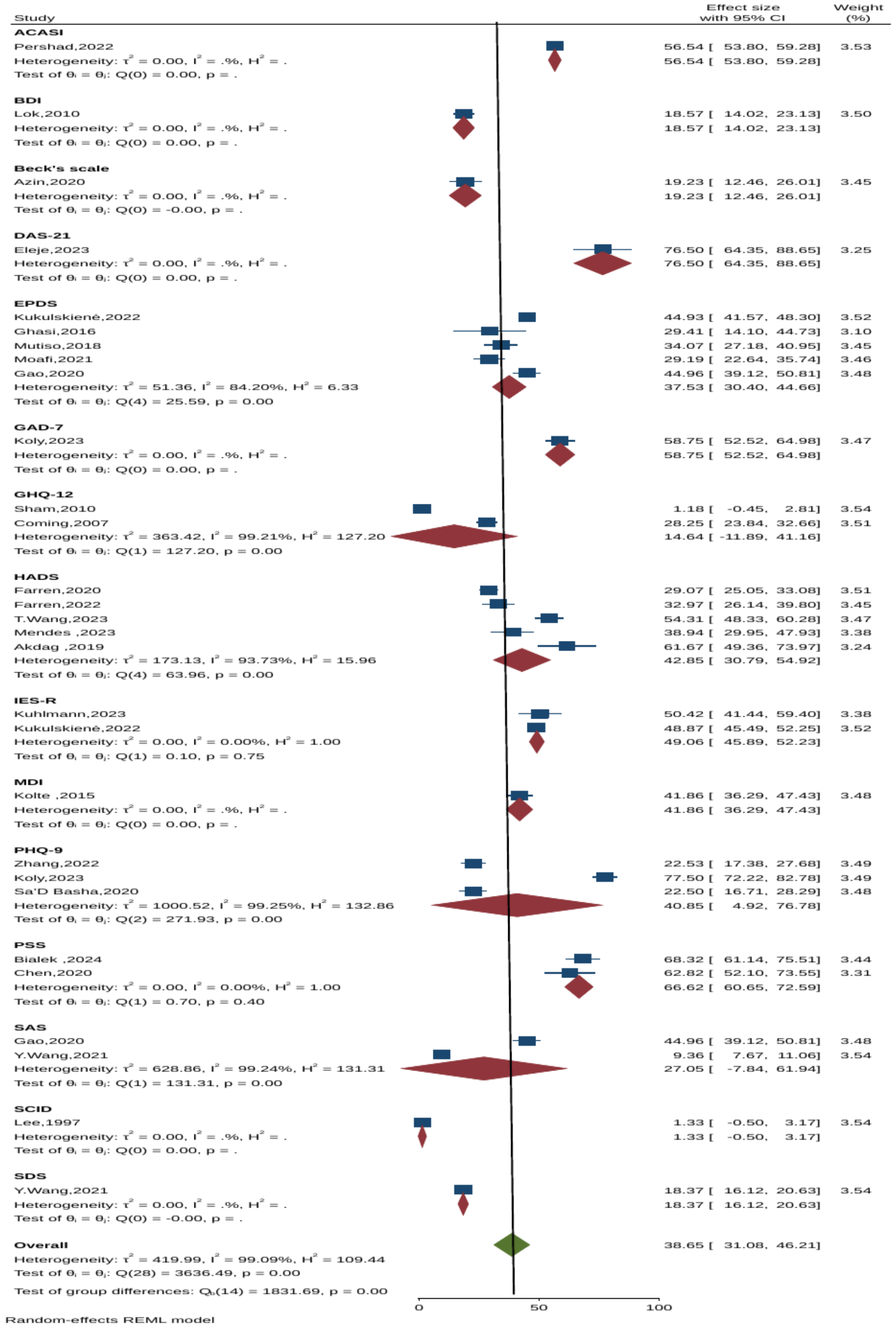

Figure S5. Different standardised scales used to measure anxiety, depression, and stress.

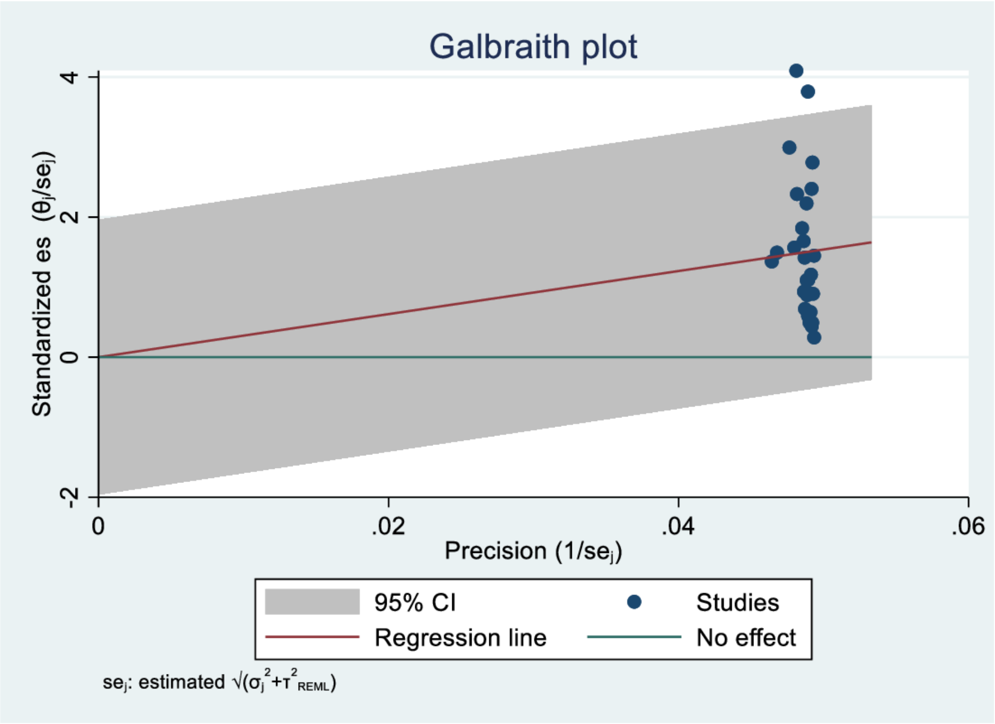

Figure S6. Galbraith plots explaining the study-specific effect sizes, precisions, the overall effect size, and detecting potential outliers.

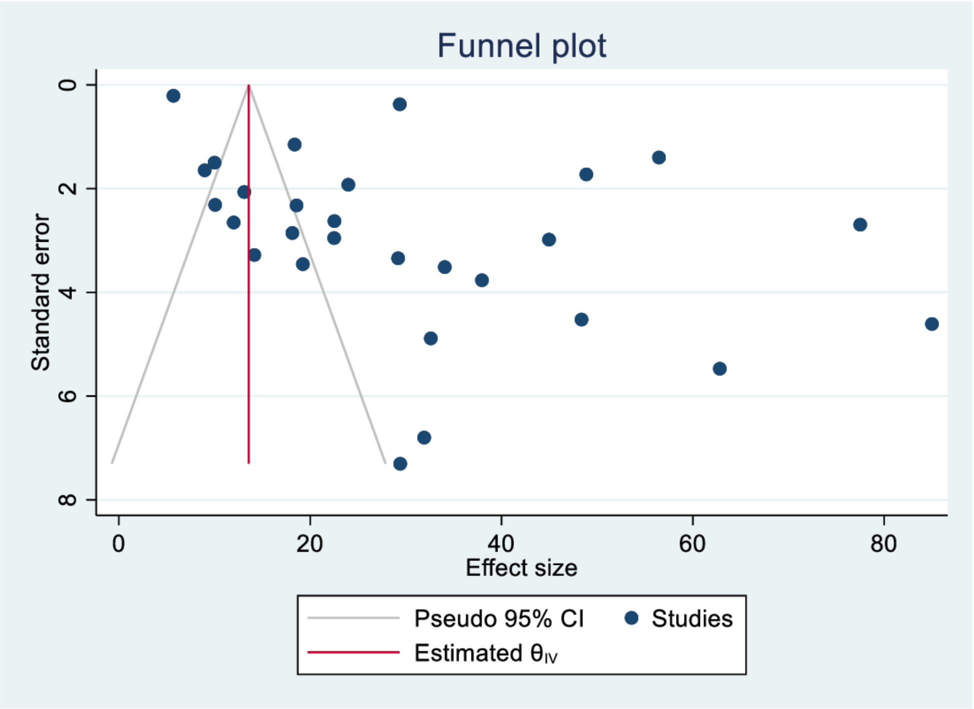

Figure S7. Funnel plot of publication bias of included studies.
